# Cellular Immune Response Induced by mRNA Vaccines Against SARS‐CoV‐2

**DOI:** 10.1002/iid3.70375

**Published:** 2026-03-11

**Authors:** Valentina Tovar, Isaura Pilar Sánchez, María Teresa Rugeles, Natalia Andrea Taborda, Juan C. Hernandez

**Affiliations:** ^1^ Grupo Inmunovirología, Facultad de Medicina Universidad de Antioquia Medellín Colombia; ^2^ Grupo de Investigaciones Biomédicas Uniremington, Programa de Medicina, Facultad de Ciencias de la Salud Corporación Universitaria Remington Medellín Colombia; ^3^ Infettare, Facultad de Medicina Universidad Cooperativa de Colombia Medellín Colombia

**Keywords:** cancer, cellular immunity, COVID‐19, mRNA vaccines, organ transplant, SARS‐CoV‐2

## Abstract

The disease caused by SARS‐CoV‐2 is known as COVID‐19, and it can range from mild symptoms to severe clinical manifestations, including respiratory failure, pneumonia, and organ failure. Since its emergence in 2019, more than 7 million deaths have been reported worldwide. Vaccines have been the most effective strategy for preventing severe illness and death in patients who acquire the infection. Vaccines induce both humoral and cell‐mediated immune responses; the latter is crucial in the immune response against SARS‐CoV‐2, as the effector mechanisms of T‐cells are less affected by the high mutation rate of the virus and prevail through memory phenotypes, ensuring long‐term protection. mRNA vaccines have been primarily used worldwide to control the COVID‐19 pandemic. This platform can protect against different circulating variants and is characterized by generating a robust T‐cell response. This review discusses the immune response of T‐cells induced by mRNA vaccines against SARS‐CoV‐2. It explores their effect on different population groups, including people with special clinical conditions, such as cancer and organ transplant recipients with a compromised immune system.

## Graphical abstract

SARS‐CoV‐2 vaccine induces cellular immune response. T‐cells play a protective role for COVID‐19.

## 1 Introduction

SARS‐CoV‐2 was first reported in December 2019. By February 2026, more than 779 million confirmed cases and about 7.1 million deaths were reported worldwide [1]. The disease caused by SARS‐CoV‐2 is called “coronavirus disease 2019” (COVID‐19), and like other coronaviruses of zoonotic origin, such as SARS‐CoV and MERS‐CoV, it is associated with severe respiratory infections [2]. Its clinical presentation ranges from asymptomatic cases, mild symptoms (including dry cough, fever, fatigue, and loss of taste and smell), and severe cases, including respiratory failure, pneumonia, and organ failure, which can result in death [3].

SARS‐CoV‐2 constantly undergoes mutations due to random errors during its replication process in the host cell, recombination processes, and the selective pressure generated by the immune response and antiviral treatments [4]. Mutations in the spike (S) protein, which mediates the entry of the virus into host cells, have led to the appearance of new variants of concern (VOCs). This term refers to a viral genome that may include mutations with an impact on the characteristics of the virus, and that may increase the rate of transmission and the severity of the disease, compromising the effectiveness of immunity induced by natural infection or vaccination [5, 6]. Among the VOCs designated by the World Health Organization are the Alpha, Beta, Gamma, Delta, and Omicron variants (which include several lineages) [7]. For example, the B.1.1.529 Omicron variant was reported in November 2021 and contains more than 30 mutations in protein S, with a total of 15 mutations in the receptor‐binding domain (RBD), a region of the protein that interacts with the angiotensin‐converting enzyme 2 (ACE2) receptor on the host cell to allow entry of the virus [8]. Mutations include K417N and N501Y, which contribute to the escape of the SARS‐CoV‐2 vaccine‐induced immune response and increased infectivity [9, 10]. Since then, Omicron has spread throughout the world, increasing the number of new cases of COVID‐19, with the consequent appearance of lineages with new mutations, that is, a group of viruses derived from the same common ancestor [5, 11].

Vaccines against COVID‐19 have become the most effective strategy for preventing severe disease and death [12]. Currently, 12 vaccines are approved and categorized according to the vehicle and the nature of the antigen. It can be the inactivated SARS‐CoV‐2 virus, generating an immune response targeting multiple viral proteins, or it can be vaccines specifically directed to the SARS‐CoV‐2 spike (S) protein through an adenovirus viral vector, mRNA sequence, or the purified S protein [13]. This protein is the main target of vaccination since, in addition to mediating the entry of the virus into the host cell, it is the major antigenic determinant of the virus, with more than 90% of the neutralizing antibodies of convalescent patients directed to S protein RBD [14, 15]. However, it is also the region with the greatest variability, which limits the adaptive immune response of vaccines whose antigen is limited to this protein [13]. Particularly, although mRNA vaccines are directed to S protein, its versatility allows the rapid and efficient incorporation of new sequences for other SARS‐CoV‐2 variants with greater specificity [16], making it one of the most widely used vaccines worldwide, as it is also low‐cost. To date, only the Pfizer mRNA vaccines BioNTech (BNT162b2) and Spikevax/Moderna (mRNA‐1273) have been extended to the BA.4, BA.5, and more recently, lineages, together with Novavax, to the XBB.1.5 lineage of the Omicron variant to extend protection against COVID‐19 [17, 18, 19].

In general, COVID‐19 vaccines stimulate humoral and cellular immune responses. The humoral response generates specific antibodies against structural components of the virus that may block the virus from entering the host cell or enhance its recognition by other immune system components to promote its elimination [20]. The cellular immune response contributes to the enhancement of immune system cells overall, the maturation of B cells into plasma cells for the production of high‐affinity antibodies, the elimination of infected cells, and the development of T‐cell memory [21]. However, different studies have shown a decrease in antibodies over time following vaccination and an increase in cases associated with new variants, alarming the population and raising questions about the effectiveness of these vaccines [22]. The decrease in specific antibodies in vaccinated people does not necessarily indicate the absence of T and B cells with immunological memory capable of activating and responding rapidly to an encounter with SARS‐CoV‐2 [2]. Compared to antibodies, the T‐cell response is less affected by mutations in SARS‐CoV‐2 variants [23]. The gradual decline in the efficiency of the immune system over time, known as immunosenescence, and states of hyperinflammation or deterioration of the immune system due to the presence of diseases, highlight the importance of studying the relationship between these factors and the cellular immune response triggered by vaccines [24, 25, 26].

Additionally, factors such as age and comorbidities influence the immune system's ability to respond to vaccination [26]. However, information about the phenotypes of effector and memory lymphocytes induced by COVID‐19 vaccination is limited, as well as the understanding of the duration and specificity of the cellular immune response is lacking. This review will present the cellular immune response induced by mRNA vaccines against SARS‐CoV‐2, including studies in healthy adults and key populations such as children and patients with special conditions, such as cancer and organ transplant recipients who have a developing or compromised immune system due to the use of drugs or physical treatments, respectively.

## 2 Methodology

This narrative review was conducted between July 2023 and August 2024 to summarize the current knowledge on mRNA vaccination (BNT162b2 and mRNA‐1273) in children, cancer patients, and organ transplant recipients. The literature search was performed in Google Scholar and PubMed using the following keyword combinations: COVID‐19 AND/OR mRNA vaccination, BNT162b2 OR mRNA‐1273; COVID‐19 AND/OR mRNA vaccination, BNT162b2 OR mRNA‐1273 AND children; COVID‐19 AND/OR mRNA vaccination, BNT162b2 OR mRNA‐1273 AND cancer; COVID‐19 AND/OR mRNA vaccination, BNT162b2 OR mRNA‐1273 AND organ recipients.

The inclusion criteria were studies published in English from 2021 onward. Studies involving animal experimentation were excluded. Relevant articles were selected based on their contribution to understanding the immunological response, safety, and efficacy of mRNA COVID‐19 vaccines in the specified populations.

## 3 SARS‐CoV‐2 Pathogenesis and Immune Response Against Natural Infection

SARS‐CoV‐2 is a positive‐sense single‐stranded RNA virus belonging to the *Coronaviridae* family. It has structural proteins that include a membrane glycoprotein (M) and a glycoprotein called spike (S), anchored to the lipid envelope (E). The envelope encapsulates a helical nucleocapsid protein (N) that binds to viral RNA [8]. The spike protein mediates virus entry into host cells through receptor‐mediated endocytosis of ACE2, which is predominantly expressed in the plasma membrane of epithelial cells that line the airways, especially in the alveolar cells of the lungs [27]. This protein consists of two subunits: the S1 subunit contains the RBD, which interacts directly with the ACE2 receptor to allow virus entry, and the S2 subunit, which is involved in the fusion of the virus envelope with the cell membrane. When RBD interacts with ACE2, the spike protein undergoes a conformational change that exposes a hydrophobic region in the S2 subunit called the “fusion peptide.” This fusion peptide inserts into the cell membrane of the host cell, allowing the fusion of both membranes and facilitating the entry of the virus into the cell [8].

In the nasal epithelium, the viral RNA is recognized by Toll‐like receptors (TLR3, TLR7, or TLR8 in the endosome) and by the retinoic acid‐inducible gene‐I (RIG‐I) in the cytoplasm [27]. Signaling after the activation of these receptors results in the translocation of transcription factors, such as nuclear factor kappa‐B (NF‐kB) and the consequent transcription of antiviral and proinflammatory cytokines to limit the spread of SARS‐CoV‐2 through the respiratory tract [28, 29]. When the effector mechanisms of the innate immune system are insufficient to halt viral spread, SARS‐CoV‐2 can disseminate to the alveoli and other organs, which is associated with severe stages of the disease, leading to a cytokine storm with significant increases in proinflammatory cytokines like IL‐6, IL‐8, IL‐10, IL‐1RA, TNF‐α, and CXCL10 [30].

When adaptive immunity is activated, neutralizing antibodies against the spike protein block the binding and entry of the virus into host cells [31, 32]. In addition, CD4^+^ and CD8^+^ T‐cells produce cytokines that enhance the effector mechanisms of other cells and eliminate infected cells by releasing granzymes and perforins or through FAS/FASL apoptosis, respectively [31, 33, 34]. Previous studies have highlighted the importance of cellular immunity both during SARS‐CoV‐2 infection and in the post‐recovery phase, highlighting the relevance of memory T‐cells in the control of infection and prevention of the development of severe illness from future infections [35, 36, 37, 38]. CD4^+^ T‐cells are classified into subpopulations, including Th1, Th2, Th17, and follicular helper (Tfh) [28]. All these are essential during both the initiation and the effector phases of innate and adaptive immune responses; particularly, Tfh cells secrete IL‐21 in germinal centers, which is essential for promoting survival, proliferation, and B‐cell isotype switching to produce high‐affinity antibodies [39, 40]. Although SARS‐CoV‐2 mainly induces a Th1 response, which is associated with high production of IFN‐γ [40, 41], an increased frequency of Th2 and Th17 cells has also been detected in COVID‐19 patients, particularly those who develop severe illness or die [40, 42]. Still, its role in this disease is unclear [35, 43]. Through the adaptive immune response, central (T_CM_) and effector (T_EM_) memory cells are generated [44, 45, 46]. Both phenotypes have been detected in convalescent COVID‐19 patients and vaccinated people after in vitro stimulation of peripheral blood mononuclear cells with SARS‐CoV‐2‐specific peptides. These cells demonstrated their effector capacity by releasing IFN‐γ, which induces a specific memory response over time [46, 47, 48]. On the other hand, SARS‐CoV‐2‐specific memory CD4^+^ T‐cells and CD8^+^ T‐cells have also been detected in healthy people without previous infection, which indicates that the cross‐recognition of T‐cells derived from previous encounters with other endemic coronaviruses [2, 45, 49]. Additionally, tissue‐resident memory CD4^+^ and CD8^+^ T‐cells (T_RM_) have been found in the lungs and the respiratory tract. These cells have the potential to rapidly exert their effector functions upon re‐encountering the virus in convalescent patients and are present at very low frequencies in vaccinated individuals without prior exposure to the infection [50, 51, 52]. Like T_EM_, T_RM_ do not express CCR7, which has posed a challenge in distinguishing between the two phenotypes. Currently, CD69, whose expression is induced following T‐cell activation, and CD103, an alpha E integrin expressed on mucosa‐associated T‐cells, are used as markers for T_RM_ recognition [53]. Together, these T‐cell phenotypes allow the evaluation of the magnitude and durability of the cellular immune response to infection and vaccination [38]. Notably, although T‐cells do not directly prevent the establishment of infection, cellular immunity reduces severe cases and deaths from COVID‐19. A reduction in T‐cells or a change in their cellular phenotype caused by primary or secondary immunodeficiencies, age, the presence of other infections, or immunosuppressive treatments can affect their effector function and the production of high‐affinity antibodies [39].

## 4 Immune Response Induced by mRNA Vaccines

**Figure 1:**
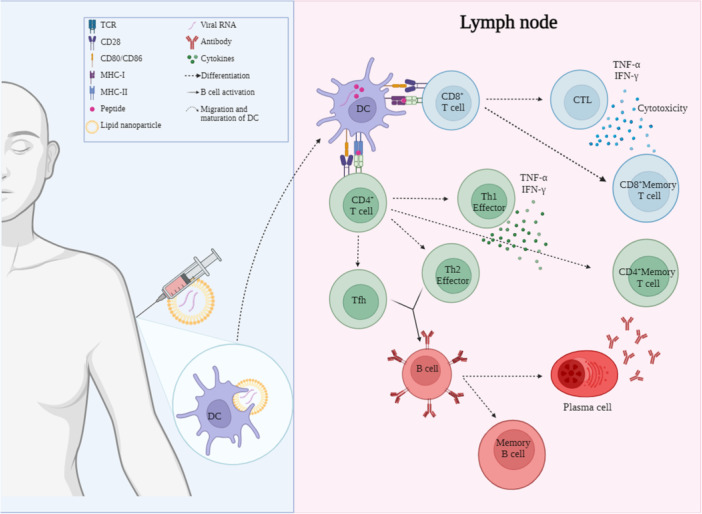
Immune response induced by vaccination with mRNA against SARS‐CoV‐2. The recognition of lipid nanoparticles by dendritic cells, their migration to secondary lymphoid organs, and the development of the adaptive immune response are illustrated, highlighting the differentiation and function of T and B cells.

At the injection site, lipid nanoparticles that encapsulate mRNA encoding the spike protein of SARS‐CoV‐2 are internalized by local antigen‐presenting cells (APCs). The encoding mRNA is released into the cytosol of APCs and, as in natural infection, is recognized by TLRs and RIG‐I, which activate innate immunity [54, 55]. The mRNA encoding the spike protein is translated into the antigen (S protein) by ribosomes in APCs. The resulting polypeptides undergo processing in the proteasome, leading to their presentation in the context of MHC‐I molecules on the surface of APCs. This process activates CD8^+^ T‐cells [54, 56]. Conversely, antigen presentation via MHC‐II occurs through the uptake of exogenous proteins released by APCs, as well as through cross‐presentation facilitated by specialized dendritic cells [56, 57]. APCs migrate from the vaccination site to peripheral lymphoid organs, where antigen recognition occurs by CD4^+^ and CD8^+^ T‐cells. This favors the differentiation of naive CD4^+^ cells toward Th1, Th2, and Th17 phenotypes, and of CD8^+^ cells toward cytotoxic cells in peripheral lymphoid organs [54, 55]. In addition, the formation of germinal centers is induced by Tfh for the activation and differentiation of B cells to antibody‐secreting plasma cells (Figure 1). Some antigen‐specific activated CD4^+^ and CD8^+^ T‐cells will enter the bloodstream as recirculating lymphocytes, while others will lodge in secondary lymphoid organs awaiting a future antigen encounter [58].

## 5 Cellular Immunity Induced by mRNA Vaccines in Children and Adolescents

Since the emergence of COVID‐19, epidemiological data suggest that the infection is less frequent in children and adolescents, with a prevalence between 1.1% and 22.5%, which varies by country and age range [59]. Furthermore, most cases are asymptomatic or have mild manifestations [60, 61]. This scenario changed after the emergence of VOCs associated with greater transmissibility, such as Gamma (November 2020), Delta (December 2020), and Omicron (November 2021), increasing the number of positive cases of COVID‐19 and hospitalization in children and adolescents, where vaccination coverage remains low [59, 62, 63]. In South Africa, an increase in mild to severe cases of COVID‐19 in minors was associated with the Omicron variant. It is important to note that, at that time in South Africa, vaccination was not approved for children under 12 [64]. Similar observations were done in Chicago, where children infected with the Gamma and Omicron variants were associated with greater severity, especially in those without immunization or previous infection by SARS‐CoV‐2 [63]. Vaccination against SARS‐CoV‐2 and a previous COVID‐19 infection helped prevent severe outcomes during the Omicron surge. The Spikevax/Moderna and Pfizer/BioNTech mRNA vaccines were the first to be licensed in children from 6 months of age by the FDA due to their safety and effectiveness in reducing virus transmission and inducing a protective humoral and cellular immune response against SARS‐CoV‐2 and its variants [65, 66, 67, 68].

As for cellular immunity triggered by mRNA vaccines, a study evaluated the frequency of specific T cells targeting the spike protein of the Wuhan variant and assessed their cross‐reactivity against the Omicron variant in children aged 5–11 years. This assessment was conducted before their first BNT162b2 vaccine and 7–15 days after receiving the second dose, following a homologous vaccination schedule. The results showed a significant increase in T‐cells producing IFN‐γ Wuhan variant after the second dose of the vaccine. Even though T‐cells exhibited cross‐reactivity against the Omicron variant, the response was more robust with a higher frequency of specific T‐cells targeting the Wuhan variant compared to the Omicron variant, which may be because the vaccine is designed to head the ancestral variant [69]. In adolescents (11–17 years old) vaccinated with a homologous regimen of three doses of BNT162b, the response of IFN‐γ and IL‐2 positive CD4^+^ and CD8^+^ T‐cells specific to the spike protein variants Wuhan and Omicron BA.1 is significantly higher post‐booster dose compared to pre‐booster, with a predominance of CD8^+^ T‐cells with powerful cross‐reactivity against Ómicron BA.1. An increase in CD8^+^(IFN‐γ^+^IL‐2^+^) T‐cells was also detected in adults [70]. A recent study assessing children aged 5–11 years who received two doses of BNT162b2 showed that those with prior SARS‐CoV‐2 infection exhibited a more robust cellular immune response compared to infection‐naïve children [71]. Specifically, virus‐experienced children demonstrated significantly higher frequencies of SARS‐CoV‐2 spike‐specific CD4⁺ T cells, as well as a greater proportion of IFN‐γ‐producing T‐cells, both 1 and 6 months after vaccination [71]. These results indicate that previous infection enhances the magnitude and persistence of vaccine‐induced T‐cell responses, supporting the concept of hybrid immunity as a contributor to long‐term cellular protection.

When comparing BNT162b2 or CoronaVac platforms, the response of spike‐specific CD8^+^ T‐cells is strong with both vaccines. Still, the production of IL‐2 in CD4^+^ T cells is weaker in those vaccinated with CoronaVac. However, both vaccination platforms induced a potent spike‐specific T‐cell response. The response to the N and M proteins also showed strong induction of CD4^+^ and CD8^+^ (IFN‐γ^+^ and/or IL‐2^+^) T‐cells in both groups. However, comparing the specific immunogenicity results against N and M between vaccination platforms is not presented because BNT162b2 does not induce an immune response against these proteins [72].

Regarding the immunocompromised pediatric population after organ transplantation, due to the use of immunosuppressive drugs to prevent rejection, studies of cellular immunity are limited. Children between 5 and 17 years who underwent solid organ transplantation exhibited an increased SARS‐CoV‐2‐specific IFN‐γ response between the second and third dose of RNA vaccination. The mean IFN‐γ concentration increased from 2.26 IU/mL after the second to 4.10 IU/mL after the third dose [73]. In a similar context, a pilot observational study assessed the humoral and cellular immune responses following two doses of the BNT162b2 mRNA vaccine in children aged 5–12 years undergoing cancer treatment [74]. The study found that 81.8% of patients with solid tumors (ST) and 85.7% of hematopoietic stem cell transplantation recipients exhibited a specific T‐cell response, whereas only 55.5% of those with hematological malignancies (HM) demonstrated such a response. Notably, 33.3% of HM and 18.2% of ST patients failed to mount any immune response, highlighting the variability in vaccine‐induced cellular immunity among immunocompromised pediatric patients [74].

In children who received kidney transplants, the frequency of spike‐specific T‐cells was lower than in patients on dialysis and healthy children, indicating the need for one or more booster doses in immunosuppressed groups [75]. Overall, the BNT162b2 vaccine induced a robust T‐cell response in the pediatric transplant population, but additional studies are needed to establish the necessity of busters.

## 6 The Response in Adults Vaccinated With mRNA Platforms

The study of cellular immunity triggered by the complete mRNA vaccination scheme (two doses) in adults has shown a long‐lasting and specific response to SARS‐CoV‐2 [76]. Phenotypically, it has been observed that mRNA vaccination preferentially induces antigen‐specific circulating follicular helper cells (cTfh), which express CXCR5 and assist B cells in generating high‐affinity antibody responses by promoting isotype switching for effective humoral response [77, 78]. Furthermore, this vaccination platform stimulates the differentiation of CD4^+^ T‐cells into the Th1 subpopulation and, to a lesser extent, into the Th2 and Th17 subpopulations [76, 79]. It has also been shown that hybrid immunity (previous infection + vaccination) is more powerful than the immune response of individuals without a history of infection and who are vaccinated, and even the response varies depending on the severity of the disease, being more robust in patients with greater severity of COVID‐19 [80, 81, 82].

Regarding the effector mechanisms of T‐cells, a study in immunocompetent individuals without evidence of previous infection reported that at 9 months after completing the vaccination, 18% of the participants exhibited IFN‐γ‐producing T‐cell responses upon stimulation with specific SARS‐CoV‐2 peptides. When the same test was performed 3 weeks after the booster dose, a significant increase in the IFN‐γ response of 64% was detected. Additionally, specific memory T‐cells from stimulation with antigenic peptides were identified, and it was found that all participants showed antigen‐specific T‐cells through AIM CD40L^+^ CD69^+^ and CD137^+^ OX40^+^ for CD4^+^ T‐cells and CD137^+^ OX40^+^ for CD8^+^ T‐cells, indicating the development of memory T‐cells after the BNT162b2 booster dose. However, the short time elapsed since the booster dose does not allow us to establish whether the memory T‐cells last over time [83]. In addition, other studies have reported a robust immune response of CD4^+^ T‐cells specific to the spike protein from the first dose, independent of previous exposure to the virus [84]. The response was highlighted mainly by the induction of T_CM_ and T_EM_ cells, as well as the high frequency of the antigen‐specific Tfh and Th1 phenotypes [76, 84].

In contrast, the emergence of CD8^+^ cells specific to the spike protein was gradual within the vaccination scheme, with greater variability in the response magnitude in individuals without previous infection [84]. The variability in the response of CD8^+^ T‐cells in the vaccinated group without previous infection in this study can be explained by the absence of viral replication in this group, which is essential for the development of memory‐specific CD8^+^ T‐cells in high responders due to an unknown previous exposure to SARS‐CoV‐2, or even to other endemic coronaviruses. In summary, these results demonstrate the robust induction of SARS‐CoV‐2‐specific T‐cells by mRNA vaccination, with qualitatively similar responses in both groups, contributing to protective immunity.

However, other studies have reported decreased cellular immunity following complete mRNA vaccination schedules [76, 85]. In this sense, a decrease in the peak maximum response of T‐cells has been observed 3 months after mRNA vaccination, in a phenomenon known as the contraction phase. After this phase, antigen‐specific memory CD4^+^ T‐cell frequencies stabilized until the sixth month after vaccination. These CD4^+^ T‐cell responses were composed of the T_CM_ (CD45RA^−^CCR7^+^) and T_EM_ (CD45RA^−^CCR7^−^) phenotypes, both in vaccinated individuals without prior exposure to SARS‐CoV‐2 and in recovered vaccinated individuals. During the contraction phase, the number of antigen‐specific T_CMM_ CCR7^+^ cells markedly decreased in the circulation, while the number of T_EM_ CCR7^−^ cells remained stable between Months 3 and 6 postvaccination.

Regarding the CD4^+^ T‐cell subsets, vaccination preferably induced antigen‐specific cTfh and Th1 cells. Despite stabilizing the frequency of specific CD4^+^ T‐cells after the third month after vaccination, the number of cTfh cells decreased rapidly, which could reflect the redistribution of the cTfh cells in the lymphoid tissues.

In contrast, antigen‐specific memory CD8^+^ T‐cells continued to decline over time after the contraction phase, with an insufficient frequency for classification into memory subsets [76]. Studies found similar results related to the production of IFN‐γ by T‐cells specific to the spike protein [76], with a decrease of about 42% in the frequency of antigen‐specific CD4^+^ and CD8^+^ T‐cells between Months 3 and 6 after completing the vaccination schedule [85].

Regarding the cellular immunity after vaccination for SARS‐CoV‐2 variants, a study compared the cellular immune response of vaccinated individuals without a history of previous infection and infected individuals without a history of vaccination [80, 81, 86]. Concerning the Wuhan variant, the response of CD8^+^ (TNF‐α^+^/IFN‐γ^+^) T‐cells was detectable in all participants, even in the control group (not vaccinated and without previous infection). This result suggests a possible infection with endemic coronavirus or SARS‐CoV‐2 infection not detected in some individuals without vaccination or previous exposure. To determine whether the Alpha and Delta variants evade the cellular immunity induced by vaccination, the cellular immune response to specific peptides of the spike protein was evaluated in each group of individuals. In this case, no significant reduction was observed in the response of CD4^+^(IL‐2^+^/TNF‐α^+^) and CD8^+^(TNF‐α^+^/IFN‐γ^+^) T‐cells compared to the response to peptides of the ancestral variant of the virus. In this case, the immunological memory induced by natural infection and vaccination against SARS‐CoV‐2 produces similar responses against the Alpha and Delta variants [81]. The participation of CD4^+^ T‐cells as the main effectors against SARS‐CoV‐2 infection has also been demonstrated when comparing the response of specific T‐cells against different variants of SARS‐CoV‐2. Interestingly, a study evaluated the cellular immune response through the intracellular detection of cytokines and ELISPOT for IFN‐γ against the Wuhan and Delta variant (B.1.617.2) in individuals vaccinated with mRNA, adenoviral vector vaccines, or a combination of both without previous infection and individuals with a history of hybrid immunity [80]. A dominant CD4^+^ IFN‐γ response was observed in SARS‐CoV‐2‐infected patients and vaccinated BNT162b2 individuals [87]. In contrast, the spike‐specific CD8^+^(IFN‐γ^+^) T‐cell response was below the detection limit in vaccinated individuals without prior infection but was high in individuals with hybrid immunity. Furthermore, the spike‐specific response of CD4^+^ and CD8^+^ T‐cells was lower for the Delta variant compared to Wuhan in both groups [80]. Another study evaluated the long‐term safety and effectiveness of the mRNA‐1273 vaccine, including a 50 µg booster dose [87]. During the Omicron BA.1 wave, a higher incidence of COVID‐19 and severe cases was observed compared to the Delta wave. Importantly, the administration of the booster dose was associated with a significant 47.0% reduction (95% CI: 39.0%–53.9%) in COVID‐19 incidence during Omicron BA.1 predominance [87]. These findings underscore the importance of booster doses in maintaining immune protection against emerging SARS‐CoV‐2 variants.

Concerning the cellular immunity of the mucosa, specifically in bronchoalveolar fluid samples, infection by SARS‐CoV‐2 triggers the formation of memory of CD4^+^ and CD8^+^ T‐cells to the spike protein in the respiratory tract. These populations exhibit antigenic specificities distinct from those of peripheral T‐cells. Although mRNA vaccination, adenoviral vector vaccines, or a combination of both also induce the generation of spike protein‐specific memory CD4^+^ and CD8^+^ T‐cells in the respiratory tract, vaccination alone does not achieve long‐lasting specific T‐cell responses in the lung mucosa against SARS‐CoV‐2. This finding supports developing vaccines specifically targeting the respiratory tract [52].

In addition to evaluating the cellular immunity induced by mRNA vaccines against SARS‐CoV‐2 and its VOCs, comparisons have been made with other heterologous vaccination regimens. In this regard, an increased IFN‐γ‐positive T‐cell response has been reported in participants who received BNT162b2 than those who received BBIBP‐CorV [88]. However, it must be considered that mRNA vaccines induce responses primarily to spike‐specific antigens, the region most susceptible to mutation. In contrast, BBIBP‐CorV induces a broader immune response targeting other structural proteins, such as N and M. Therefore, the pattern of the T‐cell response induced by BBIBP‐CorV in participants without prior infection was similar to the T‐cell response observed in convalescent patients [88].

Furthermore, developing T‐cells against other structural epitopes mitigates the impact of immune escape caused by new SARS‐CoV‐2 variants, as these regions are less susceptible to mutations [89]. When the response of CD4^+^ T‐cells was analyzed in adults with prior infection and adults vaccinated via a heterologous schedule, where all participants received at least one mRNA vaccine, CD4^+^ T_EM_ and T_CM_ cells were observed [86]. Spike‐specific CD4^+^ T‐cell response was significantly greater in individuals with hybrid immunity, as discussed previously in this manuscript and consistent with other studies [81]. The predominance of CD4^+^ T_CM_ cell phenotype (CD45RA^−^CCR7^+^) could contribute to longer‐lasting CD4^+^ T‐cell responses and antibody production by B cells. Additionally, activation of CD4^+^ T‐cells against spike peptides from Gamma and Mu variants demonstrates cross‐reactivity that protects against studied VOCs and other SARS‐CoV‐2 variants of interest, such as Mu [86].

The results suggest that mRNA vaccines protect from COVID‐19, including disease caused by VOCs such as Alpha and Delta, due to the presence of memory T‐cells secreting IFN‐γ and TNF‐α that respond to stimulation with SARS‐CoV‐2 peptides. However, the immune response to vaccination is less robust against new virus variants, which may result in the development of moderate symptomatic disease. The stronger adaptive immunity observed in vaccinated individuals who have experienced SARS‐CoV‐2 infection is due to the infection acting as a natural booster for vaccination, preventing the development of severe forms of the disease [38, 90]. However, in individuals who recovered from COVID‐19, mRNA vaccination only had a modest effect on T‐cell responses. It did not increase the frequency of memory phenotypes in spike‐specific CD4^+^ or CD8^+^ T‐cells over time. These findings underscore the need to develop new vaccines targeting circulating variants, ideally inducing mucosal memory.

## 7 Cellular Immunity in Vaccinated Adult Individuals With Cancer or Organ Recipients

People with cancer or solid organ transplants appear to be at greater risk of severe forms of COVID‐19. However, the function of the immune system in these patients may be altered (either suppressed or hyperactivated) depending on the type of disease and therapies used to treat it, making the prognosis of COVID‐19 in these patients an ongoing area of study [91]. The immunogenicity of the full vaccination BNT162b2 scheme has been evaluated in cancer patients actively undergoing immunotherapy, chemotherapy, or radiotherapy [92, 93, 94]. Regarding cellular immunity in these individuals, it was observed that in patients receiving active anti‐PD‐1/anti‐PD‐L1 therapy, the response of IFN‐γ‐producing specific T‐cells significantly decreased between the third week and sixth month after completing the vaccination schedule compared to that in immunocompetent individuals; this decline was more pronounced in cancer patients who had not experienced SARS‐CoV‐2 infection [92]. Similarly, another study evaluated the immune response to COVID‐19 mRNA vaccination in patients with CD20‐positive lymphomatous malignancies undergoing rituximab treatment [94]. While these patients exhibited diminished short‐ and long‐term IgG and IgA antibody responses compared to healthy controls, their T‐cell responses were robust and comparable to those of healthy individuals. The T‐cell response encompassed the entire spike protein, including the conserved RBD. These findings suggest that, despite compromised humoral immunity due to anti‐CD20 therapy, lymphoma patients can mount effective cellular immune responses following mRNA COVID‐19 vaccination [94].

Another study examined the impact of vaccination on three groups: patients with solid tumors, patients with hematopoietic neoplasms, and immunocompetent individuals [95]. In this research, those who completed their vaccination schedule within 21 days after the first dose were compared with those who did so later, between 70 and 77 days after the first dose. T‐cell responses were determined by IFN‐γ and IL‐2 secretion in response to stimulation with spike protein and the RBD region peptides. The frequency of cytokine‐secreting antigen‐specific T‐cell response was greater than the seroconversion rate, where dual responses (seroconversion and T‐cell response) were observed in 88% of patients with solid tumors and in only 36% of patients with hematopoietic neoplasms. There was no evidence that a delay in the second dose increased T‐cell responses, but it did enhance serological responses [95]. Other studies on patients with hematopoietic neoplasms, such as multiple myeloma and chronic lymphocytic leukemia, have also shown that there is no positive correlation between patients' serological responses and T‐cell response [96]. Failures to generate dual responses have been associated with the use of steroids, chemotherapy, or other anticancer medications within 15 days of the first and/or second dose [95]. Both studies included different types of cancer, and there were no findings related to a particular type of cancer compared to healthy patients. Patients affected by malignant hematopoietic neoplasms often present lower seroconversion rates and a lower magnitude of T‐cell‐mediated response, both through natural immunity and vaccination‐induced immunity, which can be attributed to the direct impairment of T or B cell function by the disease, damage to the bone marrow affecting their production, or direct effects of physical or chemical treatments on the number of immune system cells or their function. In another study on lymphoid malignancies by Marasco et al., in line with the results of previous research, specific SARS‐CoV‐2 T‐cell responses were detected even in the absence of seroconversion [97]. These results suggest that patients with hematological neoplasms can develop a specific cellular immune response to SARS‐CoV‐2 after vaccination. Some therapies administered during the vaccination period may alter the triggered immune response. In this regard, it would be pertinent to examine the specific effects of different treatments on vaccination efficacy, considering the complexity of the interaction between these treatments and the underlying disease.

The effect of mRNA vaccination has also been evaluated regarding booster doses in patients actively undergoing cancer treatment (immunotherapy, chemotherapy, or both). Spike‐specific T‐cells were reported in 62.3% of patients 6 months after the booster dose with BNT162b2. The mean of the highest response was observed 21 days after the third dose, confirming its ability to enhance cellular response against the Wuhan variant [93]. There is a clear need for booster doses of the mRNA COVID‐19 vaccine in cancer patients, considering that the magnitude of the cellular immune response may present greater variations in these populations compared to immunocompetent individuals due to the diversity of treatments [93]. On the other hand, in organ transplant recipients, generic medications are used to limit the immune response and prevent rejection of the transplanted organ. Consequently, the cellular immune response in these individuals is expected to be less robust compared to immunocompetent individuals and even in those with cancer. This phenomenon occurs because cancer‐targeted treatments can impact the immune system differently, adapting to each patient's specific needs.

The efficacy of mRNA vaccines has been extensively studied in individuals with renal failure receiving hemodialysis and in kidney transplant recipients [98, 99]. Most transplant patients develop a spike‐specific CD4^+^ T‐cell response, significantly lower than that in dialysis patients and healthy controls [98]. A lower frequency of IFN‐γ, TNF‐α, and IL‐2‐producing CD4^+^ and CD8^+^ T‐cells was also observed in transplant patients compared to dialysis patients and healthy controls [98]. The response of spike‐specific CD8^+^ T‐cells was lower than CD4^+^ T‐cells in hemodialysis patients and nearly undetectable in transplant patients. The analysis was performed 8 days after completing the vaccination schedule, so the durability of the cellular immune response could not be determined. Another study evaluated the effect of a booster dose of mRNA‐1273 2 months after administration in patients without prior COVID‐19 who had received lung, heart, liver, pancreas, or kidney transplants and were actively receiving immunosuppressive therapy. The frequency of spike‐specific CD4^+^(IL‐2^+^ IFN‐γ^+^) T‐cells specific to SARS‐CoV‐2 was greater in transplant patients who received the booster dose than in transplant patients who received a placebo [100]. Although the booster dose was shown to be safe in terms of side effects and increased the amount of spike‐specific CD4^+^ T‐cells, the study had a short follow‐up and did not detect differences between clinical parameters, such as the effect of immunosuppressants and type of transplant, due to the small number of patients sharing specific clinical conditions.

In general, mRNA vaccines elicit a cellular immune response against SARS‐CoV‐2 in immunocompromised patients, including solid‐organ transplant recipients. However, the magnitude of this response is significantly reduced in transplant patients compared with individuals undergoing dialysis and with immunocompetent subjects. This diminished cellular immunity highlights the need for tailored vaccination strategies in this population, including booster doses and longitudinal immune monitoring to optimize protective efficacy (Table 1).

**Table 1:** Impact of mRNA vaccines on cellular immunity.

Population/Patient groups	Impact on cellular immune response	
Adults	Response increased 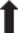	*Induces antigen‐specific CXCR5^+^ cTfh cells, which assist B cells for effective humoral response with high‐affinity antibody production [83, 84].
		*Stimulates the differentiation of CD4^+^ T‐cells into Th1, Th2, and Th17 subpopulations [82, 85].
		*IFN‐γ‐producing T‐cell responses upon stimulation with specific SARS‐CoV‐2 peptides in individuals without evidence of previous infection, at 9 months post‐vaccination. This response significantly increases (from 18% to 64%) 3 weeks after the booster dose [89].
		*Induction of antigen‐specific T‐cells CD40L^+^ CD69^+^ and CD137^+^ OX40^+^ for CD4^+^ T‐cells and CD137^+^ OX40^+^ for CD8^+^ T‐cells, indicating the development of memory T‐cells after the BNT162b2 booster dose [89]. *In vaccinated individuals with or without prior SARS‐CoV‐2 infection, a robust immune response of CD4^+^ T‐cells (T_CM,_ T_EM,_ Tfh, and Th1 phenotypes) specific to the spike protein from the first dose is observed [90]. *Response of CD4^+^ T‐cells in adults with prior infection and adults vaccinated via a heterologous schedule, where all received at least one mRNA vaccine, CD4^+^ T_EM_ and T_CM_ cells were observed. The predominance of CD4^+^ T_CM_ cell phenotype (CD45RA^−^CCR7^+^) could contribute to longer‐lasting CD4^+^ T‐cell responses and antibody production by B cells [92]. *Variability in the response of memory‐specific CD8^+^ T‐cells in a vaccinated population without previous infection can be explained by the absence of viral replication in some people [90].
	Response decreased 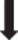	*A study revealed a decrease in response of antigen‐specific memory CD4^+^ T‐cells (CD45RA^−^CCR7^+^ T_CM_ and CD45RA^−^CCR7^−^ T_EM_) after mRNA vaccination (3 months), termed the contraction phase. After this phase, memory CD4^+^ T‐cell frequencies stabilized until the sixth month, both in individuals without prior exposure to SARS‐CoV‐2 and exposed [82]. *Despite the stabilization of the frequency of specific CD4^+^ T‐cells after the third month after vaccination, the number of cTfh cells decreased rapidly, which could reflect the redistribution of the cTfh cells in the lymphoid tissues [82]. *Decrease (42%) in antigen‐specific CD4^+^ and CD8^+^ T‐cells frequency between Months 3 and 6 after the vaccination schedule. No relationship between the decrease in the T‐cell response and age, sex, or comorbidities was observed [91].
	Responses to SARS‐CoV‐2 variants	*Response of CD4^+^(IL‐2^+^/TNF‐α^+^) and CD8^+^(TNF‐α^+^/IFN‐γ^+^) T‐cells to peptides of the spike protein Wuhan, Alpha, and Delta variants of SARS‐CoV‐2 is observed [87]. *Both the immunological memory induced by natural infection and that generated by vaccination against SARS‐CoV‐2 produce similar responses against the Alpha and Delta variants [87]. *Intracellular detection of cytokines and ELISPOT for IFN‐γ against the Wuhan and Delta variants in individuals vaccinated with mRNA showed a dominant CD4^+^ IFN‐γ^+^ response. Spike‐specific CD8^+^ IFN‐γ^+^ T‐cell response was below the detection limit of individuals without prior infection, but was high in individuals with hybrid immunity. Spike‐specific response of CD4^+^ and CD8^+^ T‐cells was lower for the Delta variant compared to Wuhan in both groups [86]. *Infection by SARS‐CoV‐2 triggers the formation of memory of CD4^+^ and CD8^+^ T‐cells to the spike protein in the respiratory tract. Although mRNA vaccination, adenoviral vector vaccines, or a combination of both also induce the generation of this response, vaccination alone does not achieve long‐lasting specific T‐cell responses in the lung mucosa against SARS‐CoV‐2 [54].
Immunosuppressed adult individuals	Cancer	*In patients receiving active anti‐PD‐1/anti‐PD‐L1 therapy, the response of IFN‐γ‐producing specific T‐cells significantly decreased between the third week and sixth month after completing the vaccination schedule compared to that in immunocompetent individuals [97]. *In Patients with solid tumors, hematopoietic neoplasms, and immunocompetent T‐cell responses determined by IFN‐γ and IL‐2 secretion in response to stimulation with spike protein and the RBD region peptides established a frequency of cytokine‐secreting antigen‐specific T‐cells was greater than the seroconversion rate. Seroconversion and T‐cell response were observed in 88% of patients with solid tumors and 36% of patients with hematopoietic neoplasms [99]. *The direct impairment of T or B cell function by the disease, damage to the bone marrow affecting their production, or direct effects of physical or chemical treatments on the number of immune system cells or their function [99]. *Patients with multiple myeloma and chronic lymphocytic leukemia have also shown that there is no positive correlation between serological and T‐cell responses. Failures to generate dual responses have been associated with the use of steroids, chemotherapy, or other anticancer medications within 15 days of the first and/or second dose of vaccination [100]. *Patients undergoing cancer treatment (immunotherapy, chemotherapy, or both). Spike‐specific T‐cells were reported in 62.3% of patients 6 months after the booster dose with BNT162b2, confirming its ability to enhance the cellular response against the Wuhan variant [98].
	Organ recipients	*Most transplant patients develop spike‐specific CD4^+^ T‐cell response, but significantly lower than dialysis patients and healthy controls. The response of spike‐specific CD8^+^ T‐cells is nearly undetectable in transplant patients. In transplant patients, a lower frequency of IFN‐γ, TNF‐α, and IL‐2‐producing CD4^+^ and CD8^+^ T‐cells was also observed [101]. *In patients without prior COVID‐19 who had received lung, heart, liver, pancreas, or kidney transplants and were actively receiving immunosuppressive therapy, the frequency of spike‐specific CD4^+^(IL‐2^+^ IFN‐γ^+^) T‐cells specific to SARS‐CoV‐2 was greater in transplant patients who received the booster dose than in transplant patients who received a placebo. The booster dose was shown to be safe in terms of side effects [100].
Children and adolescents	Homologous vaccination schedule	*In children (5–11 years old), an increase in T‐cells producing IFN‐γ specific for the spike protein of the Wuhan variant after the second dose of the BNT162b2 vaccine. *T‐cells exhibited cross‐reactivity against the Omicron variant [77]. *In adolescents (11–17 years old) vaccinated with a homologous regimen of three doses of BNT162b vaccine, the response of CD4^+^(IFN‐γ^+^ and IL‐2^+^) and CD8^+^(IFN‐γ^+^ and IL‐2^+^) cells was significantly higher post‐dose 3, with a predominance of CD8^+^ T‐cells with powerful cross‐reactivity against Ómicron BA.1 [78].
	Different vaccination platforms	*Response of T‐cells to the Wuhan variant in children after the full BNT162b2 or CoronaVac regimen demonstrated a similarly strong spike‐specific CD8^+^(IFN‐γ^+^IL‐2^+^) T‐cell response in both groups with both vaccination platforms. *Weaker spike‐specific CD4^+^(IL2^+^) T‐cells were observed in those vaccinated with CoronaVac than in those vaccinated with BNT162b2. The response to the N and M proteins also showed strong induction of CD4^+^ and CD8^+^ (IFN‐γ^+^ and/or IL‐2^+^) T‐cells for the CoronaVac vaccine [79].
Immunocompromised pediatric population	Organ recipients	*In children (5 and 17 years of age) with solid organ transplantation, an increase in SARS‐CoV‐2‐specific IFN‐γ response was observed when evaluating the response between the second (2.26 IU/mL) and third doses (4.10 IU/mL) of RNA vaccination [80]. *In children who underwent kidney transplants, children on dialysis, and healthy children, the production of IFN‐γ in spike‐specific T‐cells was lower in patients who received kidney transplants than in patients on dialysis and healthy children, indicating the need for one or more booster doses in immunosuppressed groups [81].

## 8 Conclusion

mRNA‐based vaccines have emerged as a highly promising platform in the fight against the COVID‐19 pandemic. Their rapid and scalable production, effective immunogenicity, and excellent safety profile position them as a standout option. These vaccines have demonstrated their ability to induce specific immune responses, both humoral and cellular, directed against the spike protein of SARS‐CoV‐2. With an efficacy of 95% in preventing COVID‐19 disease, including severe cases in individuals without prior infection, their impact has been significant [101, 102].

The T‐cell responses induced by vaccination with an mRNA platform have proven to be a robust tool, both in immunocompetent individuals and in immunocompromised individuals, developing even in the absence of seroconversion. Cellular immunity not only lasts over time but also maintains its ability to respond to future virus exposures thanks to the generation of diverse sets of memory T‐cells capable of rapidly responding upon encountering the virus, thereby contributing to restoring antibody titers. It is essential to highlight the importance of continuously monitoring the immune response in immunocompromised individuals to adapt vaccination protocols as needed.

## Author Contributions

**Valentina Tovar:** investigation, visualization, writing – original draft. **Isaura Pilar Sánchez:** writing – review and editing. **María Teresa Rugeles:** validation, writing – review and editing. **Natalia Andrea Taborda:** conceptualization, formal analysis, funding acquisition, project administration, supervision, writing – original draft, writing – review and editing. **Juan C. Hernandez:** conceptualization, formal analysis, investigation, project administration, supervision, writing – original draft, writing – review and editing.

## Data Availability

All data generated or analyzed during this study are included in this published article.
